# Recurrent lymphocytic myocarditis in a young male with ulcerative colitis

**DOI:** 10.1186/2047-783X-19-11

**Published:** 2014-02-27

**Authors:** Varnavas C Varnavas, Nico Reinsch, Mareike Perrey, Felix Nensa, Thomas Schlosser, Hideo A Baba, Guido Gerken, Raimund Erbel, Rolf A Janosi, Antonios Katsounas

**Affiliations:** 1 Department of Cardiology, University Hospital Essen, Hufelandstrasse 55, 45147 Essen, Germany; 2 Institute of Diagnostic and Interventional Radiology, University Hospital Essen, Hufelandstrasse 55, 45147 Essen, Germany; 3 Institute of Pathology and Neuropathology, University Hospital Essen, Hufelandstrasse 55, 45147 Essen, Germany; 4 Department of Gastroenterology and Hepatology, University Hospital Essen, Hufelandstrasse 55, 45147 Essen, Germany

**Keywords:** Ulcerative colitis, lymphocytic myocarditis, heart failure

## Abstract

Awareness of myocarditis in association with inflammatory bowel diseases is crucial as it bears a rare but serious risk for mortality. This report describes the case of a young Caucasian male, whose heart biopsy was tested negative for giant cells and bacterial or viral genomes or proteins. He was experiencing severe lymphocytic myocarditis (other than mesalamine-induced) along with cardiogenic shock during ulcerative colitis exacerbation. This is an extremely rare, if not unique, clinical constellation. We chose to study the epidemiologic grounds and all major aspects of differential pathogenesis and treatment of this serious health problem.

## Background

Awareness of myocarditis, pericarditis or myopericarditis [1] in association with inflammatory bowel diseases (IBD), e.g. ulcerative colitis (UC) and Crohn’s disease, is crucial, as they convey a rare but serious risk for mortality. The onset of symptoms often occurs during IBD exacerbation and prognosis varies from a mild cardiac disorder to severe cardiogenic shock. To date, most of the cases reported in association with IBD (other than infectious or ‘giant cell’ myocarditis), consider the myocarditis as a side effect of mesalamine therapy [2]. Lymphocytic myocarditis as a pure extraintestinal manifestation of IBD is extraordinarily rare [3].

## Case presentation

A 30-year-old Caucasian male with a one-year history of UC was admitted to our intensive care unit (ICU) due to acute heart failure from an external hospital, where he had presented with abdominal pain and bloody diarrhea two weeks previously. These symptoms were assumed to arise from UC exacerbation. Prednisone (1 mg/kg-body-weight/day) and azathioprine (2 mg/kg-body-weight/day) had been added to the standard therapy with mesalamine (4 g/day). Ten days later, the patient rapidly developed aggravated symptoms of left heart failure. A transthoracic cardiac echocardiogram (TTE) confirmed severe impairment of left ventricular (LV) function with the ejection fraction (EF) approaching 15%, before the patient was referred to our ICU.

**Figure 1:**
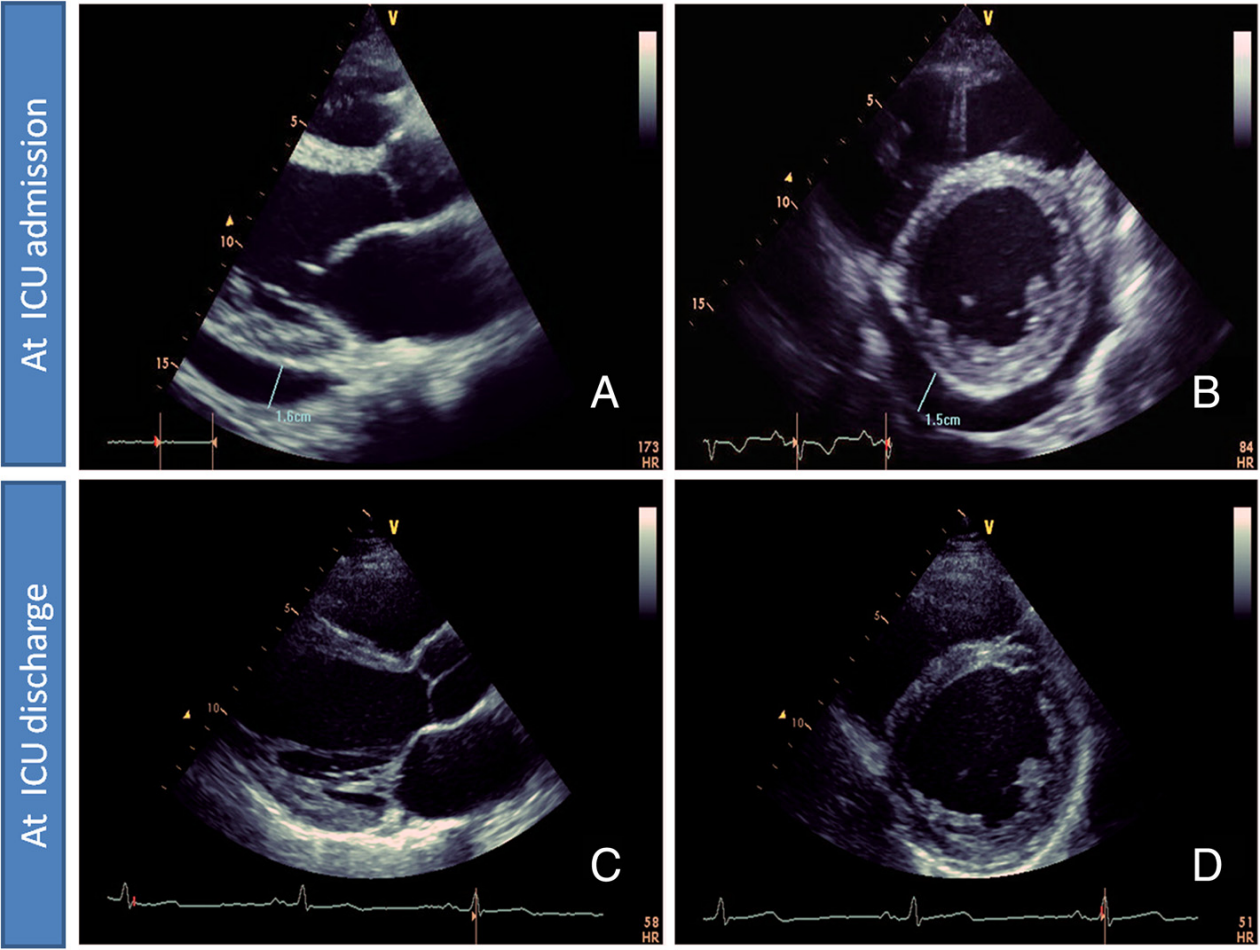
**Transthoracic cardiac echocardiogramms.** At ICU admission: **(A)** long axis view, and **(B)** short axis view; both views show a pericardial effusion of 1.6 cm. At ICU discharge: **(C)** long axis view, and **(D)** short axis view; both views confirm significant reduction of pericardial effusion.

On ICU admission, a 12-lead electrocardiogram (ECG) detected sinus tachycardia of 100/min and non-specific ST-T wave changes without any significant evidence of acute myocardial ischemia. A chest X-ray showed no pulmonary infection. TTE verified LV dysfunction with advanced hypokinesia to akinesia of the basal/middle-inferior/septal/anterior wall resulting in an EF of 13% and revealed pericardial effusion (PE) of 1.6 cm (Figure 1A,B). These findings were accompanied by abnormal laboratory tests including anemia (hemoglobin: 7.6 g/dl), leukocytosis (16.25/nl) and elevated levels of C-reactive protein (27.6 mg/dl), troponin-I (7.6 ng/ml), myoglobin (664 g/dl), brain natriuretic peptide (4744.7 pg/ml), creatinine (1.43 mg/dl), lactate dehydrogenase (565 U/l), aspartate aminotransferase (253 U/l), alanine aminotransferase (166 U/l) and gamma-glutamyl transpeptidase (119 U/l). Table 1 shows the initial as well as follow-up routine biochemistry parameters along with in-house-specific reference values.

**Table 1:** Initial and follow-up biochemistry values based on routine diagnostic tests

**Parameter**	**Initial admission**	**Initial discharge**	**Readmission**	**Final discharge**	**Reference**	**Unit**
Leucocytes (WBCs)	16.25	9.11	8.71	6.78	3.6–9.2	/nl
Red Blood Cells (RBCs)	2.63	4.14	4.97	5.08	4.5–5.6	/pl
Hemoglobin (Hb)	7.60	12.00	14.30	14.6	13.7–17	g/dl
Hematocrit (Hct)	0.22	0.37	0.42	0.421	0.4–0.5	l/l
Mean Corpuscular Volume (MCV)	82.90	89.40	83.70	82.9	83–98	fl
Mean Corpuscular Hemoglobin (MCH)	28.90	29.00	28.80	28.7	28–33	pg
Mean Corpuscular Hemoglobin Concentration (MCHC)	34.90	32.40	34.40	34.7	32–36	g/dl
Platelets	499	321	263	224	140–320	/nl
Mean Platelet Volumen (MPV)	9.00	9.20	9.70	10.2	9.4–12.2	fl
Prothrombin Time (PT or Quick)	48	96	98	97	70–130	%
International Normalized Ratio (INR)	1.45	1.04	1.02	1.02		
Activated Partial Thromboplastin Time (aPTT)	28.60	26.30	27.10	26.8	24.4–32	sec
Fibrinogen	557	448	566	386	210–400	mg/dl
Sodium	127	138	142	142	136–145	mmol/l
Potassium	5.60	4.80	4.60	4.7	3.5–5.1	mmol/l
Calcium	1.94	2.29	2.38	2.48	2.08–2.6	mmol/l
Magnesium	0.78	0.83	0.79	0.81	0.66–1.0	mmol/l
Phosphate	4.40	4.40	2.80	3.2	2.7–4.5	mg/dl
Creatinine	1.43	0.84	1.24	1.32	0.9–1.3	mg/dl
Blood Urea Nitrogen (BUN)	42.00	21.00	22.00	22	6–19.8	mg/dl
Creatinine Kinase (CK)	418	13	45	59	38–174	U/l
Creatinine Kinase –MB (CK-MB)	61				<25	U/l
Creatinine Kinase –MB% (CK-MB %)	15					%
Troponin I	7.60	0.04	0.42	0.08	0–0.1	ng/ml
Myoglobin	664	21	30	20	10–67	μg/l
Aspartat Aminotransferase (ASAT)	253	19	15	21	0– < 50	U/l
Alanine Aminotranseferase (ALAT)	166	40	28	42	<50	U/l
Gamma-glutamyl transpeptidase (GGT)	119	79	33	84	<55	U/l
Lactate dehydrogenase (LDH)	565	183	142	138	100–247	U/l
C-reactive protein (CRP)	27.60	0.70	4.10	0.6	<0.5	mg/dl
Brain natriuretic peptide (BNP)	4744.70	462.30	108.60	60.5	0–100	pg/ml
Glomerular Filtration Rate (GFR) (MDRD)	62	114	73	68		ml/min/1.73 q

Invasive blood pressure monitoring and pulmonary artery catheterization (via a Swan-Ganz catheter) were used to record hemodynamic variables. Subsequently, dobutamine (15 μg/kg body weight/minute) was administered because of deranged cardiac output (1.8 l/min) and cardiac index (0.8 l/min/m^2^). However, due to undesirable tachycardia, the dobutamine was switched to milrinone (0.75 μg/kg-body-weight/minute). On grounds of insufficient inotropic drug efficacy in line with unchanged cardiac output and cardiac index measurements and echocardiographic findings, as well as lack of clinical improvement, the use of an intra-aortic balloon pump (ECG-triggered) was considered mandatory.

While no evidence of myocardial infarction or coronary artery disease was found in the left heart, right cardiac catheterization revealed an elevated mean pulmonary artery pressure of 33 mmHg (a mean value of 15 mmHg is considered normal) and multiple myocardial biopsies were acquired.

**Figure 2:**
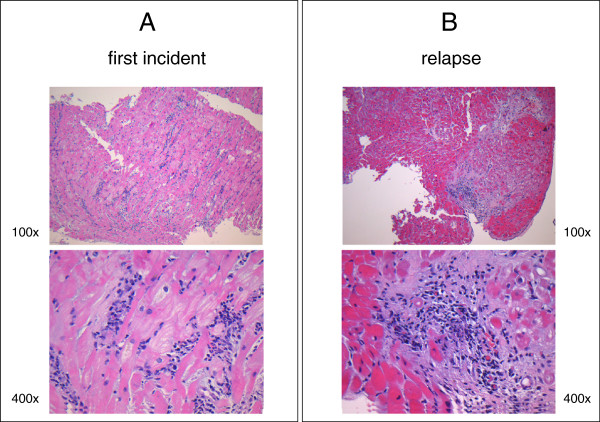
**Histology images revealed acute myocarditis with lymphocytic infiltration and moderate myocyte apoptosis at first onset (A) and relapse (B).** These biopsies did not detect giant cells or significant neutrophile infiltrates.

Histological examination revealed acute myocarditis with lymphocytic infiltration. No neutrophile granulocytes or giant cells were found (Figure 2A). Furthermore, PCR analysis of the tissue did not detect any adenovirus DNA, parvovirus B19 DNA, human herpesvirus 6 DNA, Epstein-Barr virus DNA, varicella zoster virus DNA, herpes simplex virus DNA, cytomegalovirus DNA or enterovirus RNA including Coxsackie A virus and Coxsackie B (1-6) virus. In addition, immunohistochemical analysis did not detect any cytomegalovirus-associated proteins. The patient tested negative for HIV infection based on detection of specific antibodies (via ELISA) as well as RNA (via PCR) in serum.

In consideration of the patient’s critical condition and the persistently high levels of serum inflammatory markers, the patient was started on empiric antibiotic coverage consisting of intravenous piperacillin/tazobactam (3 × 4.5 g/day over 10 days).

While awaiting systemic infection (diagnostic) results, azathioprine was paused. Further immunological and infection parameters pertinent to myocarditis were measured: rheumatoid factor (negative, <10.3 IU/ml) and antinuclear and anti-centromere antibody levels (ANA negative, HEP2-IFT <1:80; c-ANCA and p-ANCA negative, ANCA-IFT <1:10). All blood cultures collected within 3 days of admission were negative for bacterial and fungal growth.

Nine days later, the intra-aortic balloon pump was removed after gradual tapering off of milrinone accompanied by normalization of hemodynamic parameters, e.g. cardiac output was 8 l/min and cardiac index was 3.7 l/min/m^2^. At day 15 of ICU treatment, a TTE confirmed improved LV function with an EF of 50% as well as elimination of the PE (Figure 1C, D) and the patient was discharged to a ward. At this point, cardiac magnetic resonance imaging (MRI) was performed, which confirmed myocarditis along with tissue edema and moderate PE (0.3 cm); however, no late enhancement was detected. Subsequently, the heart failure therapy, i.e. bisoprolol (10 mg/day), ramipril (2.5 mg/day) and spironolactone (25 mg/day), was adjusted in a stepwise fashion based on hemodynamic parameters and clinical improvement and the patient was discharged after full recovery to ambulant treatment. Of note, the systemic therapy with mesalamine was never interrupted. Because of the active colitis, additional systemic therapy with acetylsalicylic acid was not taken into consideration after discharge from the hospital.

**Figure 3:**
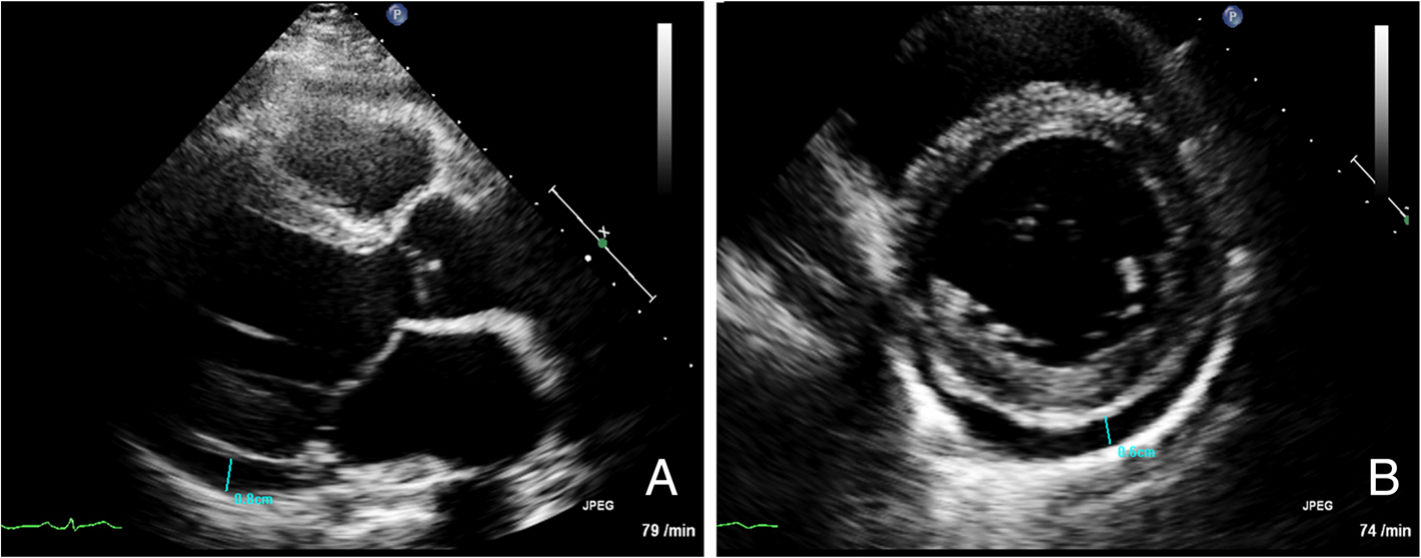
**Transthoracic cardiac echocardiogramms. (A)** Long axis view. **(B)** Short axis view. These views show a pericardial effusion of 0.8 cm at readmission.

**Figure 4:**
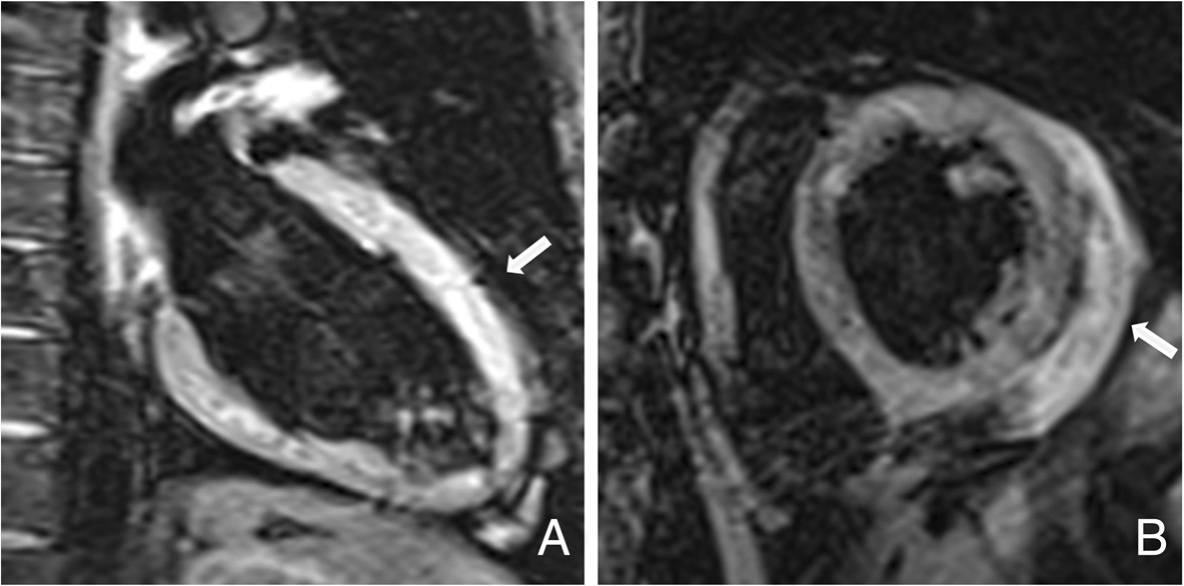
**Cardiac magnetic resonance imaging (MRI), turbo inversion recovery magnitude (TIRM) sequence. (A)** Edema in anterior wall. **(B)** Pericardial effusion of 1.2 cm.

**Figure 5:**
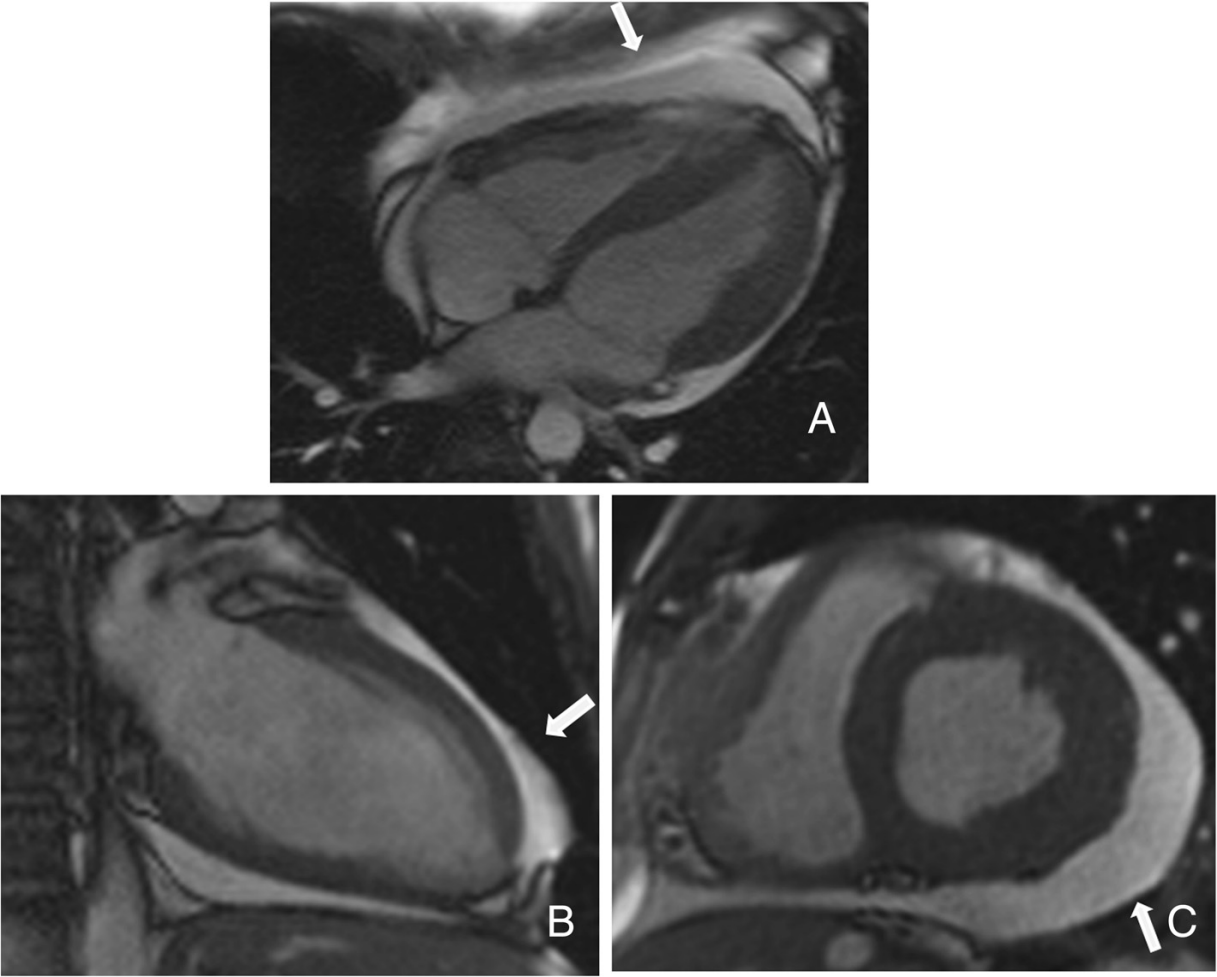
**Cardiac magnetic resonance imaging (MRI), balanced steady-state free precesion (bSSFP) sequence. (A-C)** These images show pericardial effusion of 1.2 cm.

**Figure 6:**
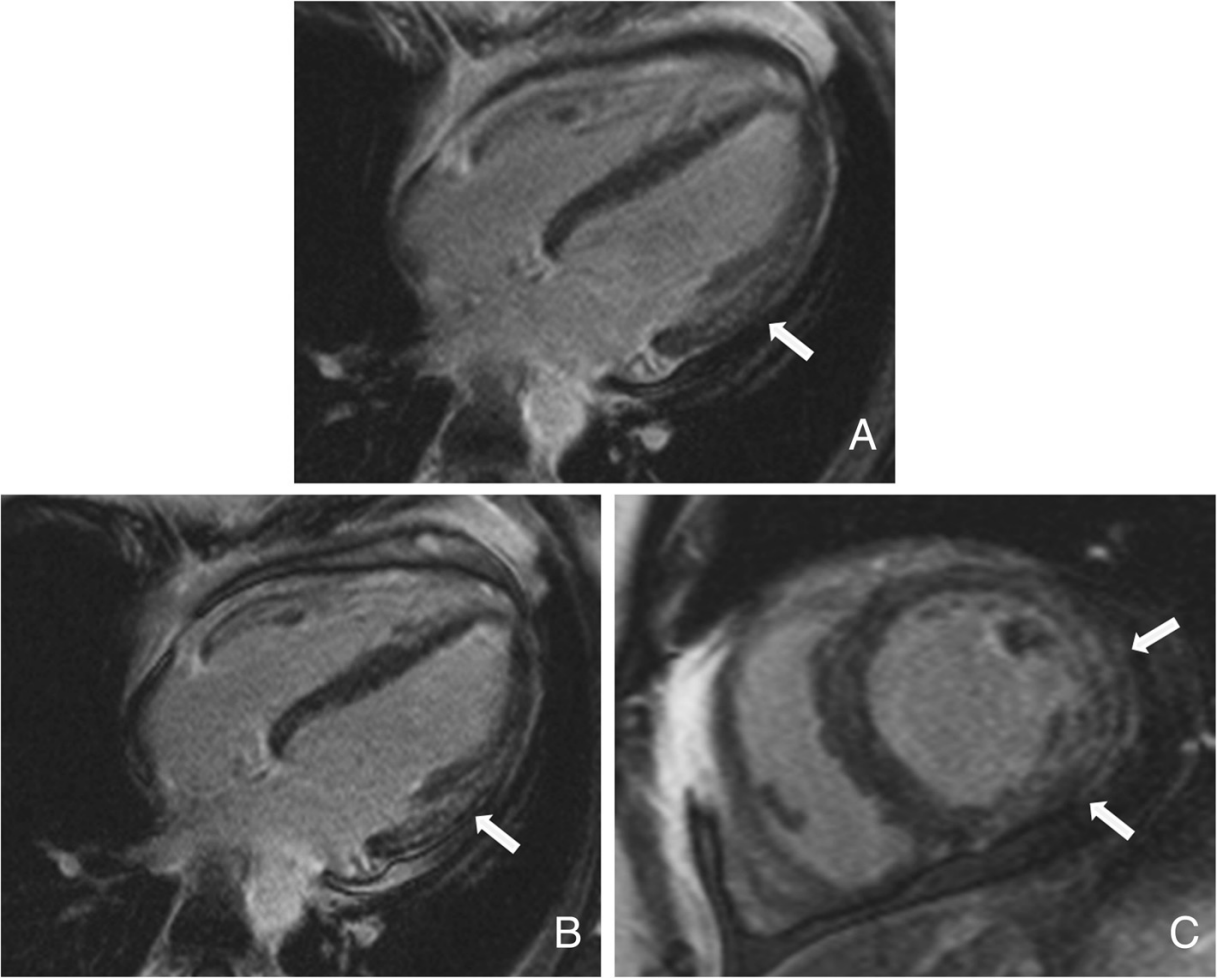
**Cardiac magnetic resonance imaging (MRI), inversion recovery (IR) turbo fast low-angle shot (FLASH) sequence. (A)** Beginning enhancement in lateral wall. **(B-C)** Intramural late-enhancement in lateral wall.

Six months after the first incident, the patient presented to our hospital with mild dyspnea on exertion and slight abdominal pain without bloody diarrhea. On admission, medication consisted of mesalamine (4 g/day) and budesonide (rectal foam, 2 mg/day). His blood pressure levels and serial ECG reports were normal. An X-ray showed no pulmonary infection. A TTE revealed a stable LV function (EF 50%); however, moderate PE (0.8 cm) was observed (Figure 3). Laboratory tests demonstrated slight elevations of inflammatory parameters and brain natriuretic peptide (Table 1). Thus, we promptly assumed that the beginning of an UC re-exacerbation might have triggered recurrent myocarditis. Unfortunately, the patient declined colonoscopy. As expected, a follow-up cardiac MRI revealed anterior LV wall edema along with PE (1.2 cm) and lateral wall late enhancement, indicative of acute myocarditis (Figures 4, 5 and 6). Again, myocardial biopsies were obtained, which showed the same pattern of lymphocytic myocarditis (Figure 2B). This time, the patient’s clinical condition improved on administering prednisone (1 mg/kg-body-weight/day), bisoprolol (10 mg/day), ramipril (2.5 mg/day) and spironolactone (25 mg/day). After discharge, a 6-month follow-up TTE showed normal LV function and no PE.

## Conclusions

An association of cardiac disease with IBD has rarely been found [2]. In a large nationwide study of IBD patients (*N* = 15,572) conducted from 1977 to 1992 in Denmark, the incidence of myocarditis was 0.03% [2]. In these cases, the most frightening scenario is finding giant cells in a biopsy. Giant cell myocarditis, which often coincides with autoimmune diseases, has been reported as inevitably fatal with a fulminate course resulting in death, usually within 3 months [4-6]. To date, less than five cases have been reported in the literature of IBD-related myocarditis (other than giant cell myocarditis) that rapidly ends in severe heart failure during UC exacerbation for either new onset or relapse cases. This convinced us of the need to report this case of a young male experiencing severe lymphocytic myocarditis along with left heart failure during UC exacerbation.

**Figure 7:**
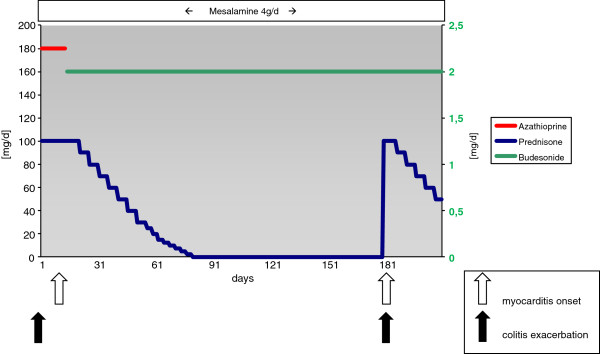
**Medication curves.** The graph illustrates dose and application time of systemic treatment with prednisone, azathioprine and mesalamine and local therapy with budesonide during the entire observation period. This graph also indicates the time points of clinical exacerbation of ulcerative colitis along with emergence of myocarditis symptoms.

Lymphocytic myocarditis emerges through an interstitial influx of lymphocytes followed by consecutive myocyte apoptosis [4]. Apparently, lymphocytic infiltrates have been observed in most cases of biopsy-verified myocarditis. Of these, the positive detection of circulating cardiac auto-antibodies and/or viral genomes in myocardial biopsy specimens or bacterial or fungal systemic infections represent the most trivial findings in patients experiencing active IBD-related lymphocytic myocarditis (reviewed in [3,4,7]). Biopsy results for our patient were negative for viral genomes or proteins. No bacterial or fungal infections were found in peripheral blood during all stays or follow-up visits in our hospital. Azathioprine was paused on first admission to the ICU as it is of unproven benefit for myocarditis other than for giant cell myocarditis [4]. Therefore, both times acute phase immunosuppressive therapy was confined to systemic therapy with prednisone [7-9]. Because of the symptoms of co-emergent severe colitis, systemic therapy with prednisone was considered mandatory. In any other case, intrapericardial treatment with triamcinolone, especially in patients with perimyocarditis, would be an equally effective local therapy option [10]. Interestingly, 6 months after the first incident, the patient’s cardiac function deteriorated after discontinuation of prednisone [7] (Figure 7). This event had clinical, TTE, MRI and histological evidence of recurrent myocarditis, which improved after recontinuation of immunosuppressive therapy and is consistent with data published elsewhere [7].

IBD-related cardiac disease may also occur as mesalamine-induced myocarditis. This form of myocarditis, where eosinophilic infiltration can be observed on a biopsy, has previously been described as a mechanism of drug hypersensitivity, and the symptoms are milder [11]. Here, mesalamine-induced myocarditis is unlikely since mesalamine administration was never interrupted. Moreover, according to the Naranjo Nomogram, it is doubtful that these episodes of myocarditis were due to mesalamine [12].

## Consent

Written informed consent was obtained from the patient for publication of this case report and the accompanying images. A copy of the written consent is available for review by the editor-in-chief of this journal.

## Abbreviations

ANA: antinuclear antibody; ANCA: anti-centromere antibody; ECG: electrocardiogram; EF: ejection fraction; ELISA: enzyme-linked immunosorbent assay; IBD: inflammatory bowel disease; LV: left ventricle; MRI: magnetic resonance imaging; PCR: polymerase chain reaction; PE: pericardial effusion; TTE: transthoracic cardiac echocardiogram; UC: ulcerative colitis.

## Competing interests

The authors report no conflicts of interest. The authors alone are responsible for the content and writing of the paper.

## Authors’ contributions

VCV wrote the manuscript. AK was involved in the drafting, editing, correction and supervising of this paper. AK, VCV, NR and RAJ diagnosed and treated the patient. MP provided the TTE images. TS and FN provided the MRI images. HAB provided the histological data and images. GG and RE assisted as experts. All authors read and approved the final manuscript.
